# Structure-based virtual screening and molecular dynamics of potential inhibitors targeting sodium-bile acid co-transporter of carcinogenic liver fluke *Clonorchis sinensis*

**DOI:** 10.1371/journal.pntd.0010909

**Published:** 2022-11-09

**Authors:** Won Gi Yoo, Fuhong Dai, Jhang Ho Pak, Sung-Jong Hong, Jin-Ho Song

**Affiliations:** 1 Department of Basic Science, Chung-Ang University College of Medicine, Seoul, Republic of Korea; 2 Department of Parasitology and Tropical Medicine, and Institute of Health Sciences, Gyeongsang National University College of Medicine, Jinju, Republic of Korea; 3 Department of Convergence Medical Science, Gyeongsang National University, Jinju, Republic of Korea; 4 Department of Parasitology, School of Biology and Basic Medical Sciences, Suzhou Medical College of Soochow University, Suzhou, Jiangsu, PR China; 5 Department of Convergence Medicine, University of Ulsan College of Medicine and Asan Institute for Life Sciences, Asan Medical Center, Seoul, Republic of Korea; 6 Convergence Research Center for Insect Vectors, Incheon National University, Incheon, Republic of Korea; 7 Department of Pharmacology, Chung-Ang University College of Medicine, Seoul, Republic of Korea; Khon Kaen University Faculty of Medicine, THAILAND

## Abstract

**Background:**

*Clonorchis sinensis* requires bile acid transporters as this fluke inhabits bile juice-filled biliary ducts, which provide an extreme environment. *Clonorchis sinensis* sodium-bile acid co-transporter (CsSBAT) is indispensable for the fluke’s survival in the final host, as it circulates taurocholate and prevents bile toxicity in the fluke; hence, it is recognized as a useful drug target.

**Methodology and principal findings:**

In the present study, using structure-based virtual screening approach, we presented inhibitor candidates targeting a bile acid-binding pocket of CsSBAT. CsSBAT models were built using tertiary structure modeling based on a bile acid transporter template (PDB ID: 3zuy and 4n7x) and were applied into AutoDock Vina for competitive docking simulation. First, potential compounds were identified from PubChem (holding more than 100,000 compounds) by applying three criteria: i) interacting more favorably with CsSBAT than with a human homolog, ii) intimate interaction to the inward- and outward-facing conformational states, iii) binding with CsSBAT preferably to natural bile acids. Second, two compounds were identified following the Lipinski’s rule of five. Third, other two compounds of molecular weight higher than 500 Da (Mr > 500 Da) were presumed to efficiently block the transporter via a feasible rational screening strategy. Of these candidates, compound 9806452 exhibited the least hepatotoxicity that may enhance drug-likeness properties.

**Conclusions:**

It is proposed that compound 9806452 act as a potential inhibitor toward CsSBAT and further studies are warranted for drug development process against clonorchiasis.

## Author summary

*Clonorchis sinensis*, the Chinese liver fluke, is a parasite infecting human. People are infected via ingesting raw freshwater fishes carrying *C*. *sinensis* metacercariae, the encysted form of larvae. Clonorchiasis is a disease caused by *C*. *sinensis*, which affects primarily the host hepatobiliary organs. World Health Organization has classified *C*. *sinensis* as a biological carcinogen inducing cholangiocarcinoma. Praziquantel has been a therapeutical choice for clonorchiasis. However, the flukes resistant to the drug have emerged due to the extensive use of it. Hence, it is necessary to develop new chemotherapeutic agents to outwit the drug-resistant worms. Sodium-dependent bile acid transporter of *C*. *sinensis* (CsSBAT) is essential for the survival of *C*. *sinensis* in the bile environment. When CsSBAT is inhibited, bile acids accumulate in the worm’s tissues and cells, which are detrimental to the survival of the worm. Structure-based virtual screening is a powerful approach for compound identification and drug development. Here, we prepared CsSBAT structures using tertiary structure modeling and refinement. We identified four putative inhibitory compounds, which competitively targeted the bile acid-binding pocket of CsSBAT. One inhibitor candidate revealed the least oral toxicity and hepatotoxicity that may enhance its druggability.

## Introduction

*Clonorchis sinensis* is a trematode parasite commonly observed in humans and is transmitted by eating raw or undercooked freshwater fish contaminated with its metacercariae [1]. The intensity and duration of the infection determine the disease complications caused by this trematode. World Health Organization has classified *C*. *sinensis* as a Group 1 biological carcinogen inducing cholangiocarcinoma in humans [2].

Praziquantel has been strongly recommended for treating trematode infections in humans including clonorchiasis [3]. Due to the extensive use of praziquantel, certain trematodes in the tropical countries have developed low sensitivity to this drug [4,5]. Hence, new chemotherapeutic agents should be developed to circumvent the low sensitivity or drug resistance of these trematodes. To date, several drug candidates for trematodiasis have been tested on animals [6–9]. Some of them are under trials for clonorchiasis patients [10].

Structure-based virtual screening (SBVS) is a powerful approach for compound identification and has recently gained immense attention in drug development [11]. Several studies have reported the effectiveness of computational approaches in discovering novel antiparasitic druggable compounds [12,13]. For *C*. *sinensis*, secretory phospholipase A_2_ enzyme [14] and 20.6-kDa tegumental protein [15] were computationally evaluated as druggable targets. However, more compounds designed by SBVS are necessary for expanding drug candidate repertoire because they can be optionally tested according to the worm burden or developmental stages of *C*. *sinensis*.

Notably, bile acids trigger physiological stimuli; however, these metabolites have detrimental effects on *C*. *sinensis* survival, as evidence suggests that the accumulated bile acids can cause toxicity to the worm’s tissues and cells [16,17]. Recent studies reported that bile components are toxic or detrimental to *C*. *sinensis* survival [7,18,19]. Under *in vitro* conditions, high concentration (> 0.005%) of bile and lithocholic acid significantly decreased the worm survival [7,18,19]. A defense system against accumulation and toxicity of bile acids is prerequisite for *C*. *sinensis* survival. In this respect, bile transporters were considered as potential targets against *C*. *sinensis*.

Apical sodium-dependent bile acid transporter (ASBT) and Na^+^-taurocholate co-transporting polypeptide (NTCP), homologs of *C*. *sinensis* sodium-bile acid cotransporter (CsSBAT), contribute to the enterohepatic circulation of bile salts in human [20]. The SBATs are druggable targets as they are essential for the bile circulation, and thus several inhibitors were developed and evaluated [21–23]. It was found that CsSBAT is essential for the survival of *C*. *sinensis* in the bile. When CsSBAT was blocked using polyacrylic acid–tetradeoxycholic acid conjugate (PATD) or *CsSBAT* was knocked down using RNAi, the bile acids were accumulated in *C*. *sinensis* adults [24]. The bile acid accumulation was detrimental and shorten the survival of *C*. *sinensis* in the bile. CsSBAT was, therefore, considered as a druggable target. Its taurocholate-binding pocket can be targeted, and further inhibition could impede its transporter function.

The tertiary structures of ASBTs in *Neisseria meningitidis* [25] and *Yersinia frederiksenii* [26] were characterized with inward and outward facing conformations. These findings have contributed greatly to understanding the mechanism of the sodium ion and bile acid transport across the cell membrane. The bacterial ASBTs have α-helices constituting 10 transmembrane domains (TMs) with short loops. In human ASBT (HsASBT), TM2 and TM5 play a major role in transporting and stabilizing sodium ions [27,28]. Both TM3 and TM4 contact with taurocholate on the outward facing (OF) cavity of the transporter, while TM6 and TM7 interact with it on the inward facing (IF) cavity [29–32].

In the present study, CsSBAT structures were reliably prepared using tertiary structure modeling and refinements. For compound screening, the Lipinski’s rule of five and the rational virtual screening strategy were employed. Here, we identified a putative inhibitory compound, which competitively targeted the taurocholate-binding pocket of CsSBAT.

## Methods

### Tertiary structure modeling and refinement

The full-length cDNA sequence of CsSBAT (Acc. No. KX756671) was retrieved from GenBank database [33]. To generate three-dimensional (3D) structure of CsSBAT, we compared the 3D structures built using different 3D modeling softwares such as Swiss-Model [34], IntFOLD [35], Phyre2 [36], RaptorX [37], HHpred [38], and I-TASSER [39]. The 3D models were then refined by two steps. First, low free-energy conformations of the 3D structure were refined by full-atomic simulations using either ModRefiner [40] or FG-MD [41]. Thereafter, backbone and side chains of the structure were refined using GalaxyRefine [42] using “both mild and aggressive relaxation” method based on repeated perturbation and overall conformational relaxation with short molecular dynamics simulations. Outward-facing (OF) and inward-facing (IF) conformations of CsSBAT were modeled using the templates *Y*. *frederiksenii* (YfASBT; PDB ID: 4n7x_A) [26] and *N*. *meningitidis* ASBT (NmASBT; PDB ID: 3zuy_A) [25], respectively.

### Quality validation and binding pockets of 3D models

Potential errors in the 3D models were evaluated using PROCHECK [43], ProSA [44], and ERRAT [45]. Residue-by-residue stereochemical quality of 3D models was verified by Ramachandran plot [46] of PROCHECK. Overall quality score was analyzed by calculating atomic coordinates of the model using ProSA with a Z-score of experimentally determined structures deposited in PDB [47]. Statistics of nonbonded atom–atom interactions were validated in comparison to a database of reliable high-resolution crystallographic structures using ERRAT [45]. All structures and protein–compound interactions were visualized using UCSF Chimera v1.14 [48] and PyMOL v2.4.1 (Schrödinger, LLC, New York, NY, USA). Disordered region was predicted using CSpritz [49]. Substrate-binding sites in CsSBAT were predicted using COACH [50], referring to the analogs with similar binding sites.

### *In silico* screening of putative inhibitors

**Fig 1:**
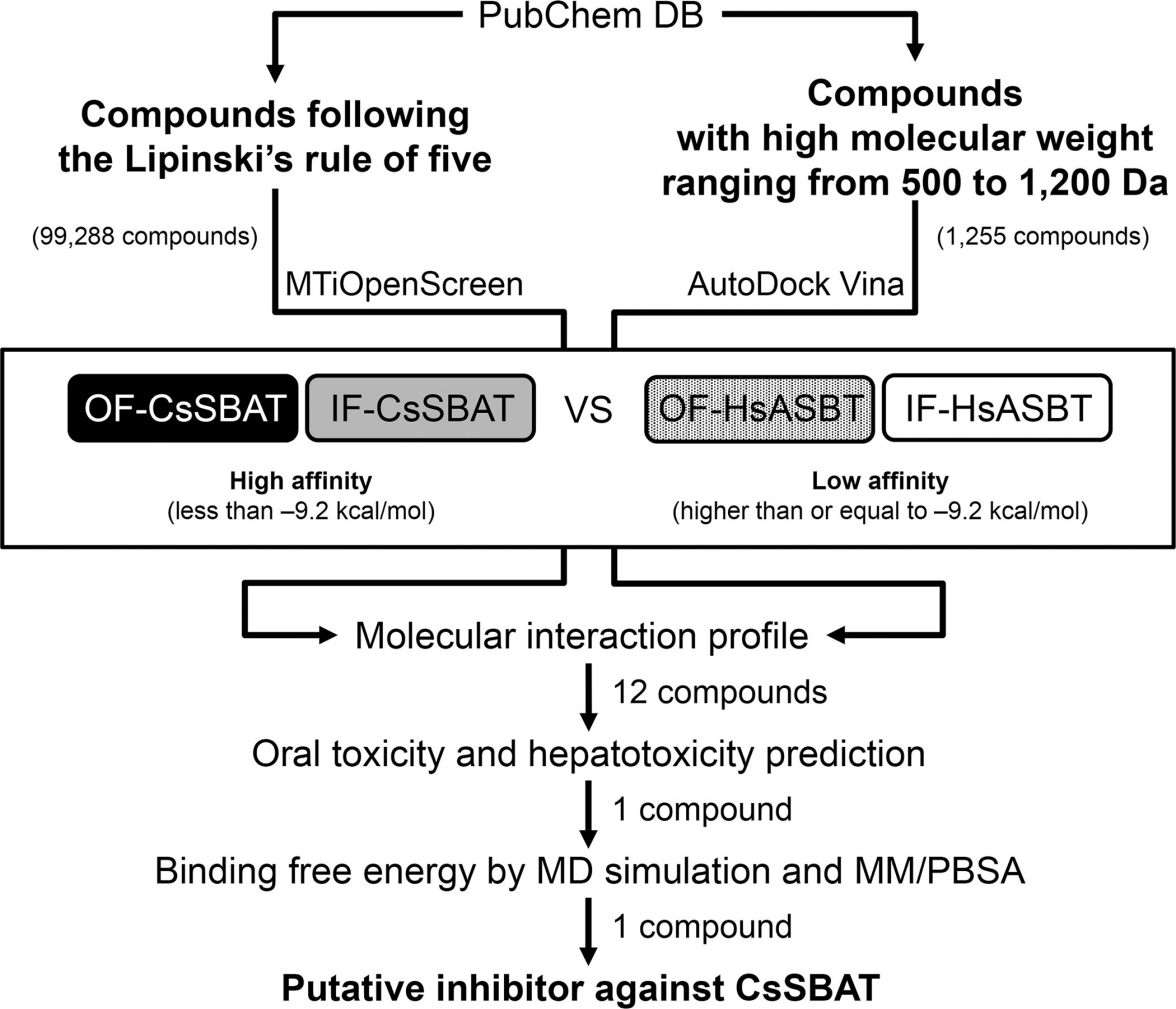
Strategy for structure-based virtual screening.

Two strategies were applied to screen the putative inhibitors against IF-/OF-CsSBAT and IF-/OF-*Homo sapiens* ASBT (HsASBT) (Fig 1): i) Compounds (99,288 ea) following the Lipinski’s rule of five [51] were screened against “diverse-lib” compounds database using MTiOpenScreen [52] as of June 2018. A list of compounds from each dataset was compared using Venn diagrams generated via jvenn [53]; ii) Compounds (1,255 ea) with high molecular weight (Mr) ranging 500–1,200 Da were collected from PubChem database [54] as of June 2018 and were screened using AutoDock Vina v1.1.2 [55]. Both docking tools were run with following parameters specifying substrate-binding pocket. These were calculated using Autoligand v1.10 [56,57]. SDF and PDBQT formats were converted into MOL2 format using Open Babel [58]. Each protein–ligand interaction map was generated using ViewDock method of UCSF Chimera v1.14 [48] and visualized using LigPlot+ v1.4.5 [59]. Massive data were analyzed iteratively and parsed using in-house Python scripts. For positive controls, bile acids (7 ea) including taurocholic acid were collected from PubChem database [54].

### Toxicity assessment

Toxicity risk and oral toxicity (LD_50_) were predicted using ProTox [60]. Higher LD_50_ dose led to less toxic compound. Toxicity class ranging 4–6 indicates that the compound is safe. Human hepatotoxicity (H-HT) was predicted using ADMETLab v2.0 [61]. H-HT value close to 1 indicates that a compound may cause liver injury.

### Molecular dynamics (MD) simulation and binding free energies

All MD simulations were performed using YASARA Structure v22.8.22 [62]. The AMBER14 force field was applied under periodic boundary conditions. The simulation cell was defined to include 20 Å surrounding the protein and filled with water, as a solvent, at a density of 0.997 g/mL. The initial energy minimization was carried out under relaxed constraints using steepest descent minimization in vacuo. All simulated systems were maintained at pH 7 by adding counter ions to replace the water containing 0.9% NaCl. Simulations were performed in water at a temperature of 298 K and constant pressure. The snapshots were saved every 250 ps for 50 ns.

Each snapshot was subjected to built-in “md_analyzebindenergy.mcr” script of YASARA Structure for the binding free energy (ΔE_bind_) calculation through Molecular mechanics/Poisson–Boltzmann surface area (MM/PBSA) using the following formula:

$$\Delta E_{bind}=[\left(E_{potProt}+E_{solvProt})+(E_{potLigand}+E_{solvLigand}\right)]–(E_{potComplex}+E_{solvComplex})$$ (1)

where E_solvProt_, E_solvLigand_, and E_solvComplex_ are the total energies of protein, ligand, and the protein-ligand complex in solvent, respectively, whereas E_potProt_, E_potLigand_, and E_potComplex_ are the total potential energies of those molecules in vacuum. The total energy is calculated as expressed below:

$$E_{total}=E_{coul}+E_{vdw}+(molsurf\times surfcost)$$ (2)

where E_coul_ is the Coulombic energy, also called electrostatic energy, E_vdw_ is the Van der Waals energy, molsurf parameter is the Van der Waals surface from the viewpoint of a solvent molecule, surfcost parameter is set to 0.65 to estimate the entropic cost of exposing an Å^2^ to the solvent. More positive ΔE_bind_ values indicate better binding. The final ΔE_bind_ values were obtained by averaging the ΔE_bind_ calculated for each snapshot of the MD simulation.

## Results and discussion

### Generation and validation of CsSBAT homology model

Both N- and C-terminal regions of CsSBAT were predicted to be hypothetical as well as disordered. In particular, disordered regions can result in long simulation time and may lead to errors in the structural clustering process [63]. Therefore, these regions were excluded from building 3D models (S1 Fig). Functional region (residues 185–492) of CsSBAT matched well with experimentally characterized ASBTs; 34.4% identity with IF-NmASBT (PDB ID: 3zuy_A) [25] and 21.4% identity with OF-YfASBT (PDB ID: 4n7x_A) [26]. Selected templates were suitable for building homology models for CsSBAT since homology model of human NTCP having 27.5% identity with NmASBT was successfully applied for analyzing new inhibitory candidates [64].

To obtain reliable homology models of the parasite protein, combined approach of 3D modeling methods and refinement were employed [15–17,65,66]. All predicted 3D models of IF-CsSBAT were evaluated using homology modeling programs such as Swiss-Model [34], IntFOLD [35], Phyre2 [36], RaptorX [37], and HHpred [38], and threading-based modeling program such as I-TASSER [39] (Table 1). Swiss-Model, IntFOLD, and HHpred revealed values greater than 91.0% in the most favored region of Ramachandran plot [46]. Except for IntFOLD presenting erroneous ERRAT value, Swiss-Model, I-TASSER, and HHpred were evaluated further in terms of refinement because I-TASSER is suitable in building structure of unaligned regions by employing *ab initio* modeling [67].

**Table 1:** Quality verifications of initial models of IF-CsSBAT according to different programs.

3D modeling program	Verification value/score			
	Ramachandran^a^	ERRAT	ProSA	Cov^b^
Swiss-Model	91.0% (0.8%)	46.0	−3.2	0.6
I-TASSER	75.8% (1.9%)	92.0	−1.1	1.0
IntFOLD	93.8% (0.2%)	n.a.^c^	−1.8	1.0
Phyre2	73.2% (5.1%)	26.9	−3.3	1.0
RaptorX	89.3% (0.4%)	41.9	−2.9	1.0
HHpred	91.7% (0.0%)	50.2	−1.4	1.0

^a^Ramachandran plot, % of most favored region (disallowed region).

^b^Cov: sequence coverage, ratio of predicted region relative to whole region.

^c^n.a.: not available.

Homology modeling of membrane proteins is challenging when the target protein shares low sequence identity (approximately 20%) with a template [68]. This issue was circumvented by refining the initial models with poor quality [15,16]. Therefore, the initial models of IF-CsSBAT were refined with ModRefiner, FG-MD, and GalaxyRefine (Table 2). After comprehensive evaluation based on the good quality of the most favored region (> 90%) and ERRAT value (> 95%), we applied Swiss-Model for reliable 3D modeling, and thereafter, FG-MD and GalaxyRefine were used for effective refinement. In fact, Swiss-Model is a powerful tool for transporter modeling [69,70]. Refining enhanced the structural quality of the final model compared to that of the initial model, particularly in terms of ERRAT values, which increased from 46.0% to 97.7% (Tables 1 and 2). However, I-TASSER and HHpred could not overcome poor values either in most favored regions of Ramachandran plot or in unacceptable ERRAT plot. Moreover, 3D models of OF-CsSBAT, OF-HsASBT, and IF-HsASBT were prepared as aforementioned.

**Table 2:** Comparison on refined structural evaluation.

3D modeling program	Refinement program			Verified score/value produced by			
	ModRefiner	FG-MD	GalaxyRefine	Ramachandran^a^	ERRAT	ProSA	Cov^b^
Swiss-Model	⚪^c^		⚪	93.3% (0.8%)	89.7	−3.8	0.6
		⚪	⚪	92.5% (1.2%)	97.7	−3.7	0.6
I-TASSER	⚪		n.a.^d^	86.8% (1.9%)	89.0	−1.1	1.0
		⚪	n.a.	74.7% (1.9%)	92.9	−1.2	1.0
HHpred	⚪		n.a.	94.1% (0.0%)	69.2	−1.9	1.0
		⚪	n.a.	76.0% (1.3%)	81.5	−1.9	1.0

^a^Ramachandran plot, % of most favored region (disallowed region).

^b^Cov: sequence coverage, ratio of predicted region relative to whole region.

^c^Symbol “⚪”: corresponding software was applied.

^d^n.a.: not available.

### Bile acid-binding cavity and sodium-binding sites

**Fig 2:**
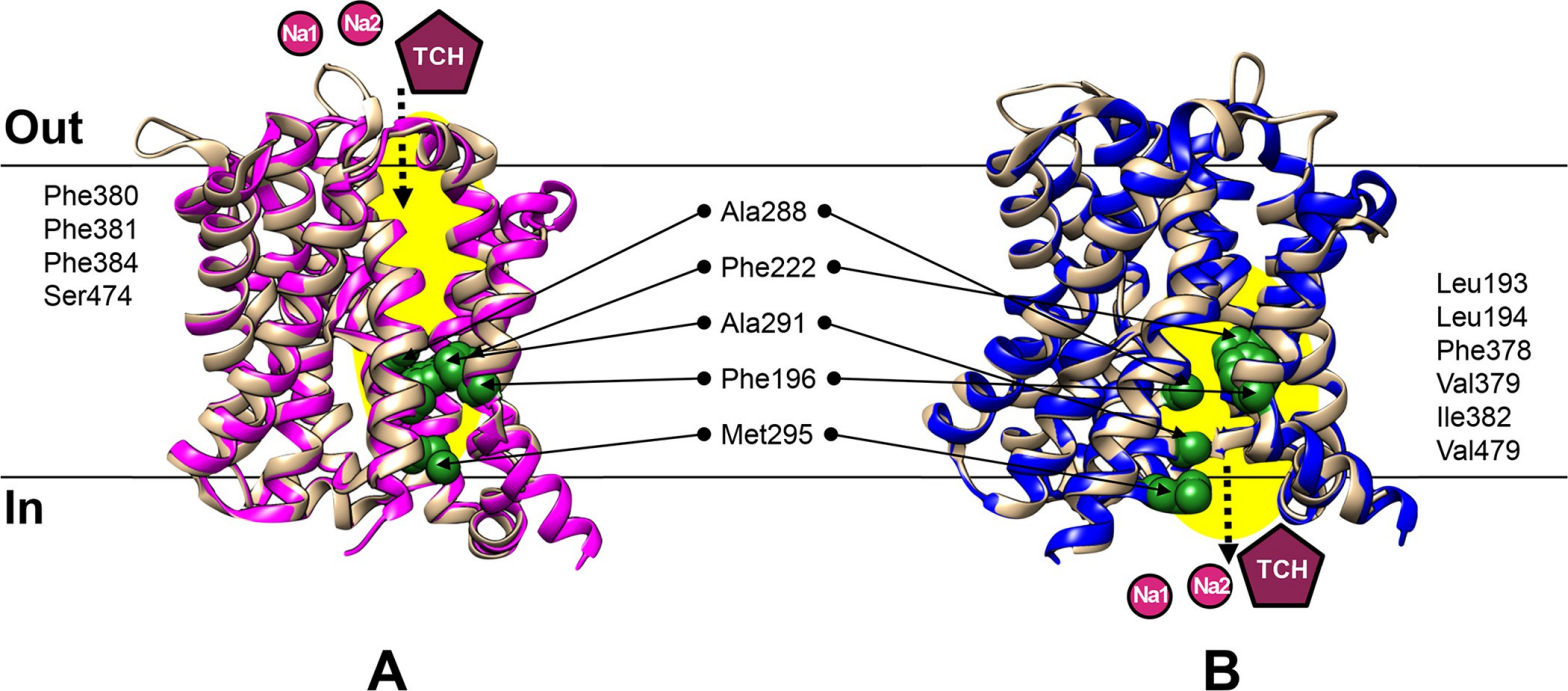
Conformation changes of CsSBAT accompanied with transport of Na^+^ and taurocholate (TCH). Both OF-CsSBAT (A) and IF-CsSBAT (B) are colored in tan. OF-YfASBT (A) is colored in pink and IF-NmASBT (B) in blue. ASBTs presenting identical conformation are superposed. Tunnel leading to the TCH-binding site is visualized in yellow eclipse. Residues forming TCH-binding cavity are depicted as green spheres in CsSBAT. Other residues participating in TCH-binding site are provided at each side. Na^+^ ions are in purple circle (Na1 and Na2). Taurocholate (TCH) is depicted as maroon pentagon.

For the translocation of bile acids across the cell membrane, alternative conformational changes in IF and OF conformations of secondary active transporters were proposed [26,71]. One of the two conformational states was observed only in a particular state because it is difficult to crystallize the structure under other states. Structural information of one state was applied to predict another conformation of transporters [72]. Here, we predicted the OF-CsSBAT and IF-CsSBAT models based on OF-YfASBT [26] and IF-NmASBT [25], respectively. In CsSBAT, the bile acid-binding pocket was presumed to be formed with 9 residues in an extracellular cavity with a volume of 908 Å^3^ in OF-CsSBAT (Fig 2A) and with 11 residues in an intracellular cavity with a volume of 986 Å^3^ in IF-CsSBAT (Fig 2B). Among them, five residues (Phe_196_, Phe_222_, Ala_288_, Ala_291_, and Met_295_) participated in pocket forming in both conformations.

ASBT is a symporter dependent on Na^+^ gradient that drives bile acid transport, which carries one bile acid with two Na^+^ ions [73]. Two Na^+^-binding sites of CsSBAT, Na1 and Na2, were predicted based on NmASBT (S2A Fig). Na1 site comprised Ser_289_, Asn_290_, Ser_303_, Thr_307_, and Glu_441_ residues, whereas Na2 site comprised Gln_252_, Glu_441_, Thr_442_, Ile_444_, and Gln_445_ residues (S2B and S2C Fig). These two sites were highly conserved with ASBTs and NTCPs [26]. Furthermore, Glu_441_ was predicted to participate in Na^+^-binding of the two sites, implying that it might have a functionally significant role (S2B and S2C Fig). In NmASBT, mutation at Glu_260_ (corresponding to Glu_441_ of CsSBAT) markedly alters the taurocholate transporting activity [25].

Recently, a putative third Na^+^-binding site was proposed, albeit rather speculative without experimental evidence [71]. The third Na^+^-binding site was reported from similar transporters such as glutamate [74] and leucine [75] transporters. In CsSBAT, residues Ile_280_, Gly_281_, Ser_283_, and Gln_445_ were predicted to act as binding sites of the third Na^+^ ion, which were superposed with the corresponding residues on NmASBT (S2D Fig). Residue Gln_445_ could be a key residue carrying Na^+^ ion from Na2 to Na3 site (S2 Fig). Mutation of Gln_258_ in YfASBT (corresponding to Gln_445_ in CsSBAT) was reported to significantly reduce the Na^+^-binding capacity of Na2 and Na3 sites [26]. Therefore, Glu_441_ and Gln_445_ in CsSBAT might act as molecular arms and transport Na^+^ ion from one site to the next.

### Putative inhibitors targeting at bile acid-binding pocket

**Fig 3:**
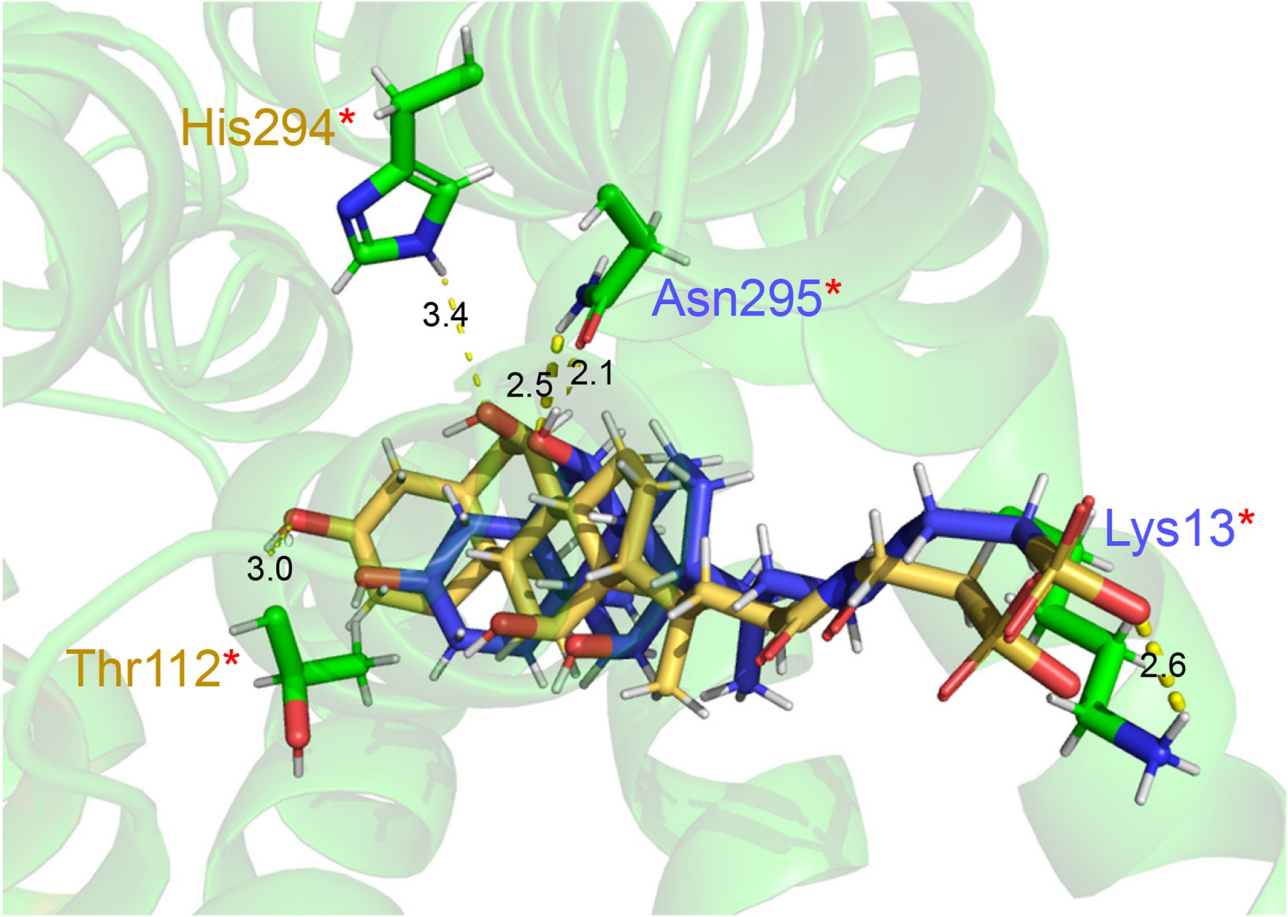
Taurocholate re-docking into the substrate-binding pocket of NmASBT. Superposition of taurocholate in the best-re-docking pose (yellow) and crystal structure (blue) (RMSD = 2.0 Å). Hydrogen bonds between the residues of NmASBT binding pocket and taurocholate in both the crystal (blue; Lys_13_ and Asn_295_) and the best-re-docked (yellow; Thr_112_ and His_294_) complex structures are shown as yellow dotted lines, and NmASBT interacting residues (green) are depicted as sticks. The known hydrogen bonds are marked in red asterisks.

For a successful large-scale virtual screening for potential compounds interacting with CsSBAT, our virtual screening protocol was validated through the re-docking of taurocholate within the binding pocket of NmASBT as suggested by Hernandez Alvarez et al. [13]. The root-mean-square deviation (RMSD) for the backbones of the re-docked pose having the best binding energies was only 2.0 Å with respect to the experimental binding mode (Fig 3). Moreover, two hydrogen bonds between taurocholate and residues, Thr_112_ and His_294,_ were reproduced in the predicted NmASBT-taurocholate complex without two Na^+^ ions (Fig 3). This result provided a reasonable prediction of experimental binding mode of taurocholate which validates our docking protocol.

For accurate molecular docking of CsSBAT against compounds in the library, grid center and size were precisely specified in the extracellular and intracellular bile acid-binding pockets of OF-CsSBAT (Fig 2A) and IF-CsSBAT (Fig 2B), respectively, rather than sodium-binding sites (S2B–S2D Fig), as described in “*In silico* screening of putative inhibitors” section of Methods.

SBVS was carried out to select putative inhibitors of CsSBAT, which satisfied the following criteria (Fig 1): i) A compound should interact more favorably with CsSBAT than with HsASBT to ensure accurate targeting. ii) OF-ASBT conformation should be considered as a target although IF-ASBT binding with taurocholate was used as a template for virtual docking [23], because the ASBTs transfer bile acid inward via conformational change from OF- to IF-conformation [76]. The Cα RMSD values between OF- and IF-conformations are 2.4 Å for CsSBAT and 1.9 Å for HsASBT. iii) The compound to be identified as a competitive inhibitor of taurocholate should reveal higher affinity than natural bile acids, ranging from −6.2 to −9.0 kcal/mol [23]. Theoretically, the binding energies of several bile acids against IF-CsSBAT and OF-CsSBAT conformations ranged from −6.1 to −8.7 kcal/mol (Table 3 and S3 Fig) [17]; however, those of HsASBT (CsSBAT homolog) were −9.0 and −9.2 kcal/mol against natural bile acids and PATD (a lead compound blocking HsASBT), respectively [23]. Thus, a cut-off value was set at −9.2 kcal/mol since the present study aimed to explore the most probable inhibitor candidates binding to CsSBAT.

**Table 3:** Binding energy for bile acids to OF-/IF-SBAT of *Clonorchis sinensis* and OF-/IF-ASBT of *Homo sapiens* using AutoDock Vina.

Bile acids	PubChem ID	*C*. *sinensis* (kcal/mol)		*H*. *sapiens* (kcal/mol)	
		OF	IF	OF	IF
Chenodeoxycholic acid	24875071	−6.7	−7.6	−7.7	−7.0
Taurochenodeoxycholic acid	312642451	−6.4	−7.7	−8.0	−8.0
Glycochenodeoxycholic acid	177011774	−6.4	−7.6	−7.7	−7.3
Glycodeoxycholic acid	57309861	n.a.^a^	n.a.	n.a.	n.a.
Deoxycholic acid	222528^b^	−6.1	−7.7	−7.3	−6.9
Taurocholic acid	828139	−6.6	−8.7	−7.1	−7.0
Glycocholic acid	177011773	−6.8	−8.5	−8.0	−6.8

^a^n.a.: not available.

^b^PubChem compound ID but other bile acids are PubChem substance ID.

### Compounds satisfying the Lipinski’s rule of five

**Fig 4:**
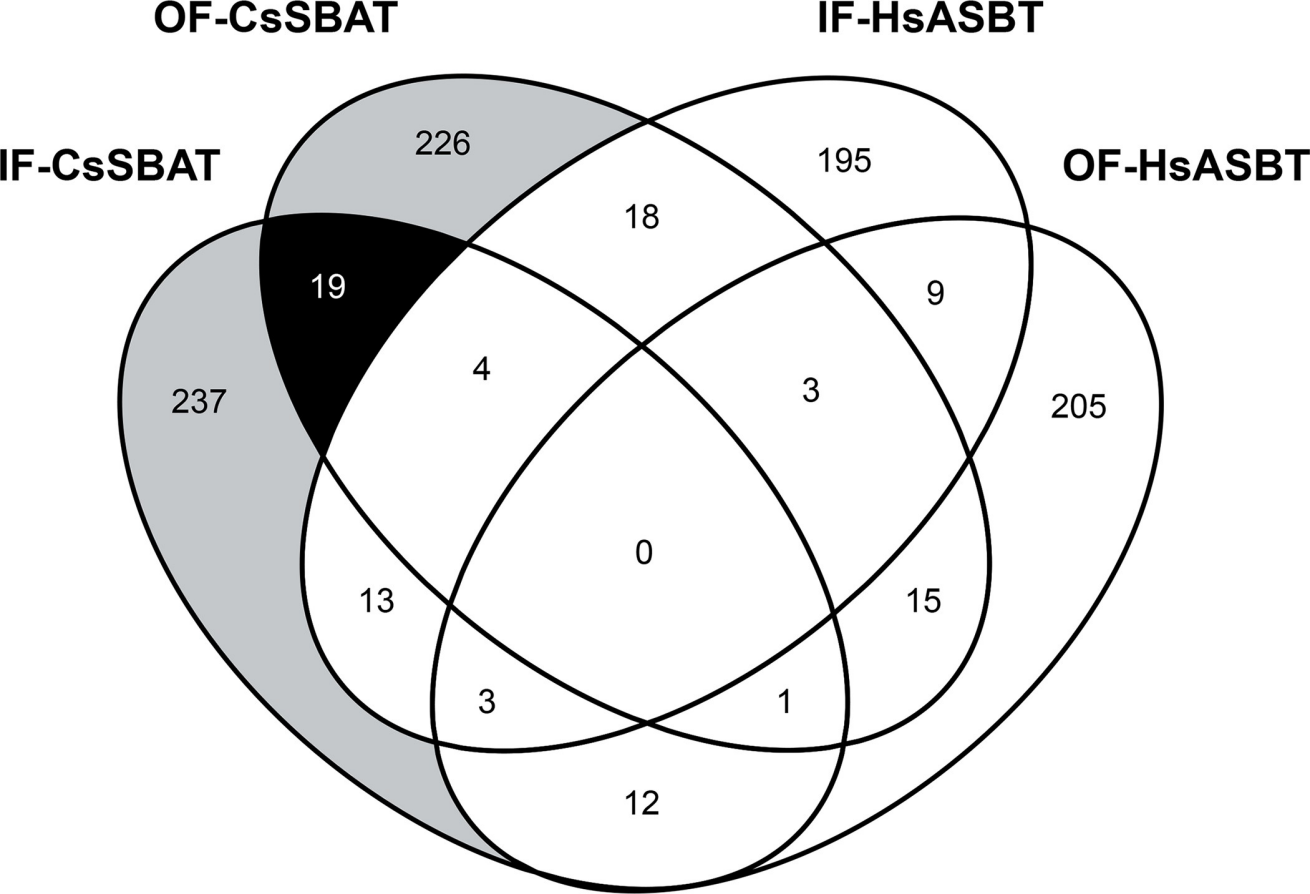
A Venn diagram presents compounds interacting with two SBATs and two ASBTs. Ellipse represents compounds screened against each of the four ASBTs. Gray area indicates compounds which interact with either OF-CsSBAT or IF-CsSBAT. Black area depicts compounds that interact with both CsSBATs, but not with other ASBTs.

**Fig 5:**
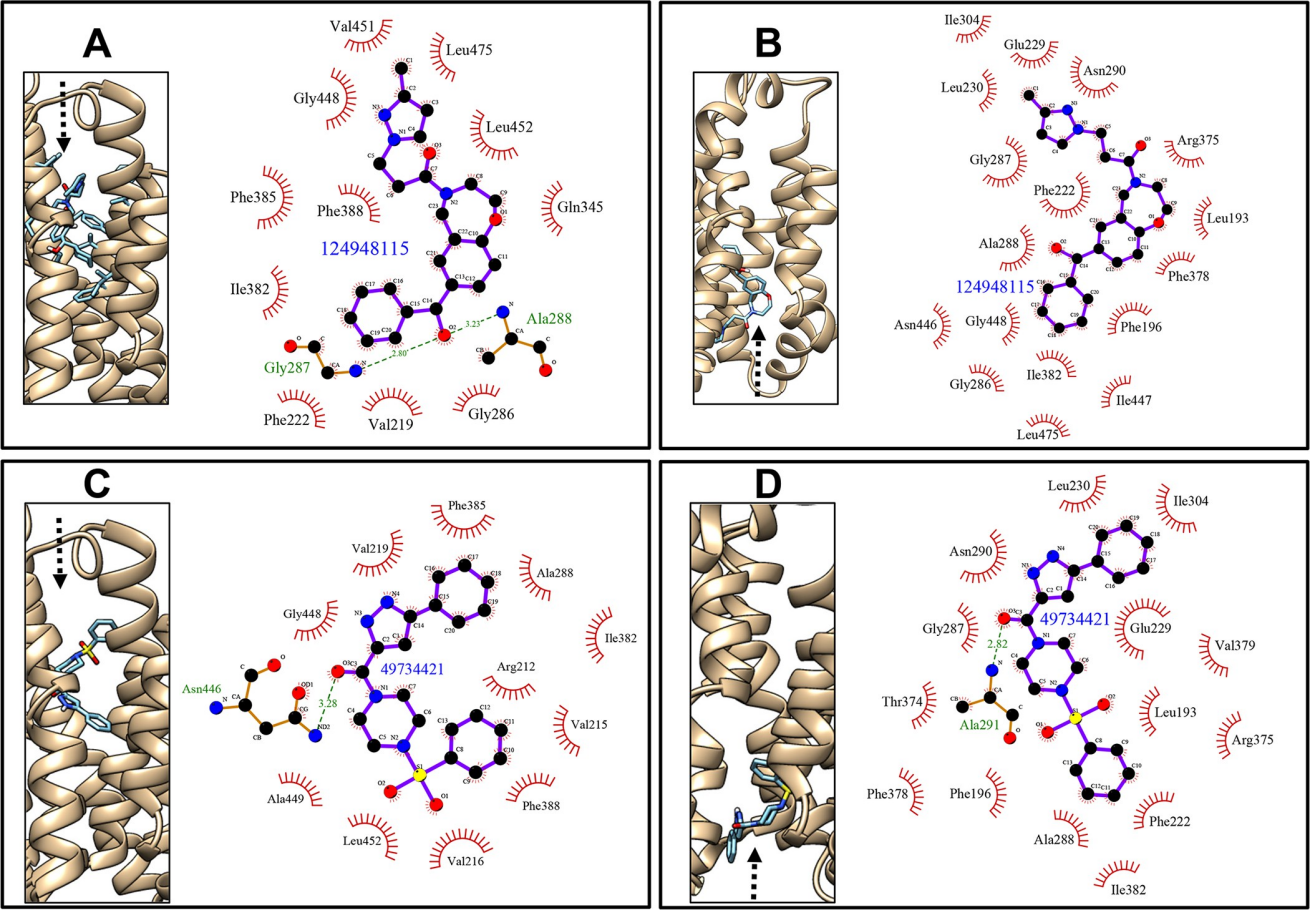
Docked pose of two compounds with IF-CsSBAT and OF-CsSBAT following the Lipinski’s rule of five. Binding modes of compounds were obtained from the MTiOpenScreen [52]. In each panel, left box ribbon and stick indicate a compound placed in the active site of OF-CsSBAT (A and C) and IF-CsSBAT (B and D). Right panel generated by LigPlot+ v1.4.3 [59] indicates the schematic interactions of CsSBAT with 124948115 (A and B) and 49734421 (C and D). The compound and residues with hydrophobic interactions are visualized with 2D diagrams. Amino acid residues involved in hydrophobic interactions are presented as red spoked arcs. Residues contributing to hydrogen bonds are depicted in green and atomic distance (Å) is given as green number. Nitrogen, oxygen, carbon, and sulfur atoms are shown in blue, red, black, and yellow, respectively. Black arrow indicates compound movement.

Compounds following the Lipinski’s rule of five [51] were screened using MTiOpenScreen [52]. Of the top 1,000 scoring compounds under docking simulation against OF- and IF-conformations of CsSBAT and HsASBT, 19 compounds that could interact with only OF-CsSBAT or IF-CsSBAT were selected (Fig 4 and S1 Table). Of these, two compounds met our strict criteria. Compound 49734421 formed a hydrogen bond with Ala_291_ of IF-CsSBAT and Asn_446_ of OF-CsSBAT. Compound 124948115 formed two hydrogen bonds with OF-CsSBAT but not with IF-CsSBAT (Table 4 and Fig 5). Majority of the residues of these two compounds were involved in hydrophobic interaction with residues on CsSBAT, implying that these interactions might play a crucial role in compound–protein interactivity. It has been reported that aromatic moieties with high hydrophobicity enable beneficial interactions with nonpolar residues in the binding pocket [77]. However, both compounds were predicted to have adverse hepatic effects, and thus their hepatotoxicity should be experimentally confirmed using human tissue and samples.

**Table 4:** Inhibitory compounds subjected to virtual screening using MTiOpenScreen and selected by applying the Lipinski’s rule of five.

PubChem ID	Binding energy (kcal/mol)		nRot	HBA	HBD	LogP	Mr	TPSA	Hydrogen bond		Toxicity class (LD_50_: mg/kg)	H-HT
	OF	IF							OF	IF		
49734421	−10.9	−9.3	4	7	1	2.3	396.5	94.8	1	1	4 (1,077)	+++ (0.95)
124948115	−10.3	−9.3	5	6	1	2.8	397.5	67.6	2	0	4 (1,500)	+++ (0.95)

Abbreviations: nRot, number of rotatable bonds; HBA, hydrogen bond acceptors; HBD, hydrogen bond donors; H-HT, human hepatotoxicity; LogP, lipophilicity; Mr, molecular weight; TPSA, topological polar surface area.

Since the Lipinski’s rule of five was introduced in 1997 [51], drug absorption or permeability has been presumed to be more likely when it has less than 5 hydrogen bond donors, less than 10 hydrogen bond acceptors, a molecular weight less than 500, and a calculated LogP smaller than 5. Recently, it was suggested that antiparasitic drugs should be exempted from this rule because several drug leads for infectious diseases do not follow the Lipinski’s rule of five [51,78]. Less stringent criteria may allow to identify more lead compounds for further assays. The suggestion motivated us to find out more effective strategy for antiparasitic drugs.

### Large compounds with high affinities

PATD was recently synthesized and evaluated as a potent inhibitor against ASBT [23]. Molecular weight of PATD is larger than 500 Da because it has several polyacrylic acids and tetradeoxycholic acids. Surprisingly, PATD is a hydrophobic substance, which violates ideal molecular weight of the Lipinski’s rule of five [51]. Nonetheless, it successfully inhibits ASBT by filling up the bile acid-binding cavity [23]. This finding motivated us to screen compounds with molecular weight larger than 500 Da, which are assumed to tightly dock CsSBAT.

Compounds of high molecular weight (500–1,200 Da) were retrieved from PubChem compound library [54] and screened using AutoDock Vina v1.1.2 [55]. Of the 1,255 compounds, 49 compounds satisfied the three given criteria. By the third criterion (higher affinity than natural bile acids), we selected 25 compounds with high affinity for CsSBAT but low affinity for HsASBT (S2 Table).

**Fig 6:**
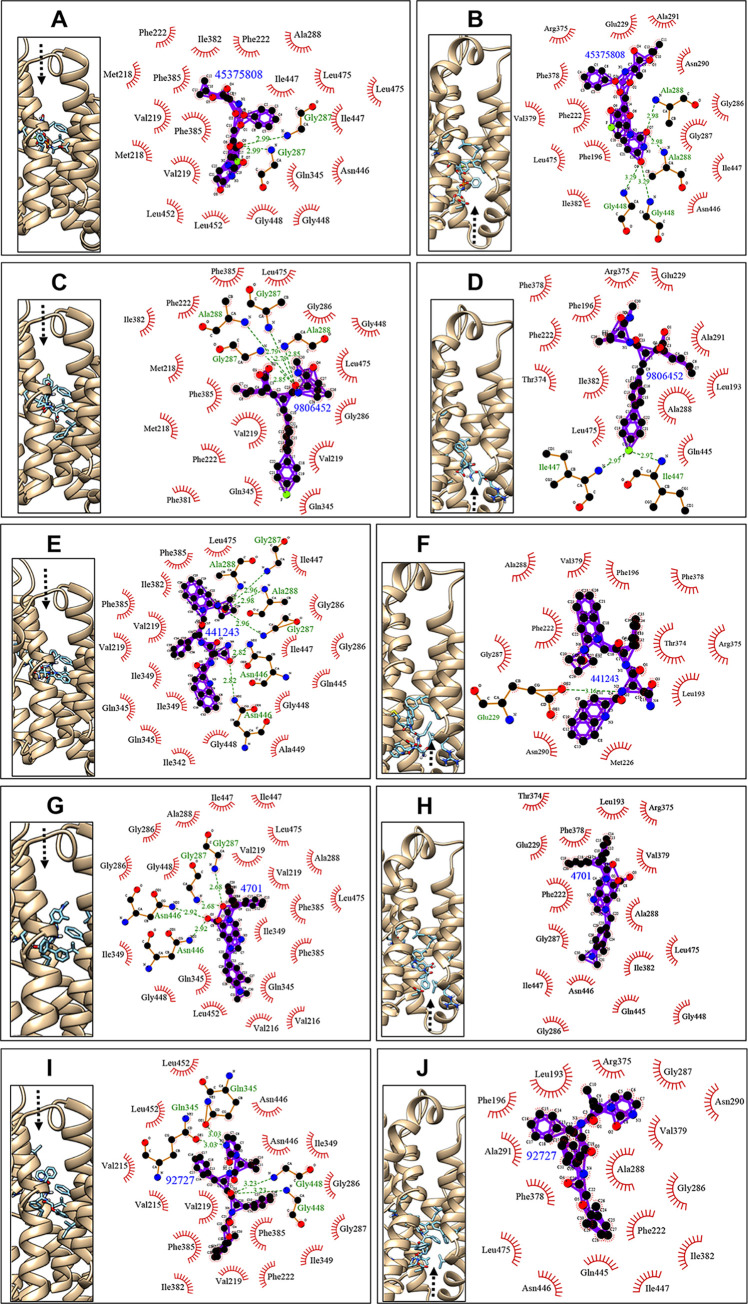
Docked poses of large compounds with OF-CsSBAT and IF-CsSBAT. The binding modes of compounds were obtained from the AutoDock Vina v1.1.2 [55]. Left (A, C, E, G, and I) and right (B, D, F, H, and J) panels represent OF-CsSBAT and IF-CsSBAT, respectively. The schematic interactions of CsSBAT with 45375808 (A and B), 9806452 (C and D), 441243 (E and F), 4701 (G and H), and 92727 (I and J) were generated using LigPlot+ v1.4.3 [59]. The compound and residues with hydrophobic interactions are visualized with 2D diagrams. The residues contributing to hydrogen bonds are colored green. Hydrogen bond is indicated as a broken line with length (Å). Amino acid residues involved in the hydrophobic interactions are marked with red spoked arcs. Black arrow indicates compound movement.

Notably, through docking simulation on compound–protein interactions, residues Glu_229_ and Gly_287_ participated in hydrogen bonding in taurocholate–IF-CsSBAT complex, whereas residues Gly_287_, Gln_345_, and Gln_348_ participated in hydrogen bonding in taurocholate–OF-CsSBAT complex (S3 Fig). Gly_287_ was involved both in taurocholate–IF-CsSBAT and taurocholate–OF-CsSBAT complexes. In majority of the compound–OF-CsSBAT complexes, this conserved residue Gly_287_ was involved in hydrogen bond interaction (Fig 6A, 6C, 6E and 6G), and Gln_345_ was involved in compound 92727–OF-CsSBAT complex formation (Fig 6I). Among compound–IF-CsSBAT complexes, only compound 441243 formed hydrogen bond with Glu_229_ (Fig 6F). Compared to taurocholate–CsSBAT complexes, Ala_288_ acted as a key residue for either hydrogen bonds or hydrophobic interactions in three compound–CsSBAT complexes (Fig 6A–6F). Compounds 441243 and 3693566 presented 5 and 6 hydrogen bonds, respectively (S2 Table); however, these compounds were excluded owing to less hydrogen bonds in compound–IF-CsSBAT interactions, which could result in off-target binding with adverse drug reactions [79].

Two compounds 45375808 (Fig 6A and 6B) and 9806452 (Fig 6C and 6D) could form more than two hydrogen bonds each with OF-CsSBAT and IF-CsSBAT without predicted toxicity (Table 5). Compound 45375808 was predicted to have drug-induced liver injury, and thus its hepatotoxicity should be validated through experimental settings.

**Table 5:** Inhibitory compounds with high molecular weight (Mr > 500 Da) selected using AutoDock Vina v1.1.2.

PubChem ID	Mr^a^	MF^b^	Binding energy (kcal/mol)				Hydrogen bond		Toxicity class (LD_50_: mg/kg)	H-HT^c^
			*C*. *sinensis*		*H*. *sapiens*					
			OF	IF	OF	IF	OF	IF		
441243	670.8	C_38_H_50_N_6_O_5_	−12.3	−10.0	−8.9	−7.9	5	1	4 (500)	+++ (0.95)
4701	508.6	C_31_H_32_N_4_O_3_	−11.2	−10.9	−8.9	−8.7	4	0	4 (800)	++ (0.82)
92727	628.8	C_37_H_48_N_4_O_5_	−10.0	−10.0	−9.0	−8.7	4	0	5 (5,000)	+ (0.69)
45375808	529.5	C_22_H_29_FN_3_O_9_P	−9.7	−9.7	−8.4	−8.4	2	4	6 (12,000)	+++ (0.95)
9806452	512.7	C_30_H_41_FN_2_O_4_	−9.3	−9.3	−7.6	−8.3	4	2	5 (3,990)	+ (0.67)

^a^Mr, molecular weight.

^b^MF, molecular formula.

^c^H-HT, human hepatotoxicity.

Compound 45375808, known as sofosbuvir, was proposed to inhibit nonstructural protein 5B polymerase in hepatitis C virus (HCV) [80]. Analogs of this compound exhibited inhibitory effect on HCV [81]. Compound 9806452 was reported as an inhibitor of matrix metalloproteinases [82] such as gelatinase A associated with tumor metastasis [83] and stromelysin-1 found in osteoarthritis [84]. Carboxyalkyl peptides containing a biphenylylethyl group inhibits adult *Schistosoma mansoni* [85]. Considering these reports, it is suggested that compound 9806452 could be an anthelminthic candidate for *C*. *sinensis*.

### Binding free energies

**Fig 7:**
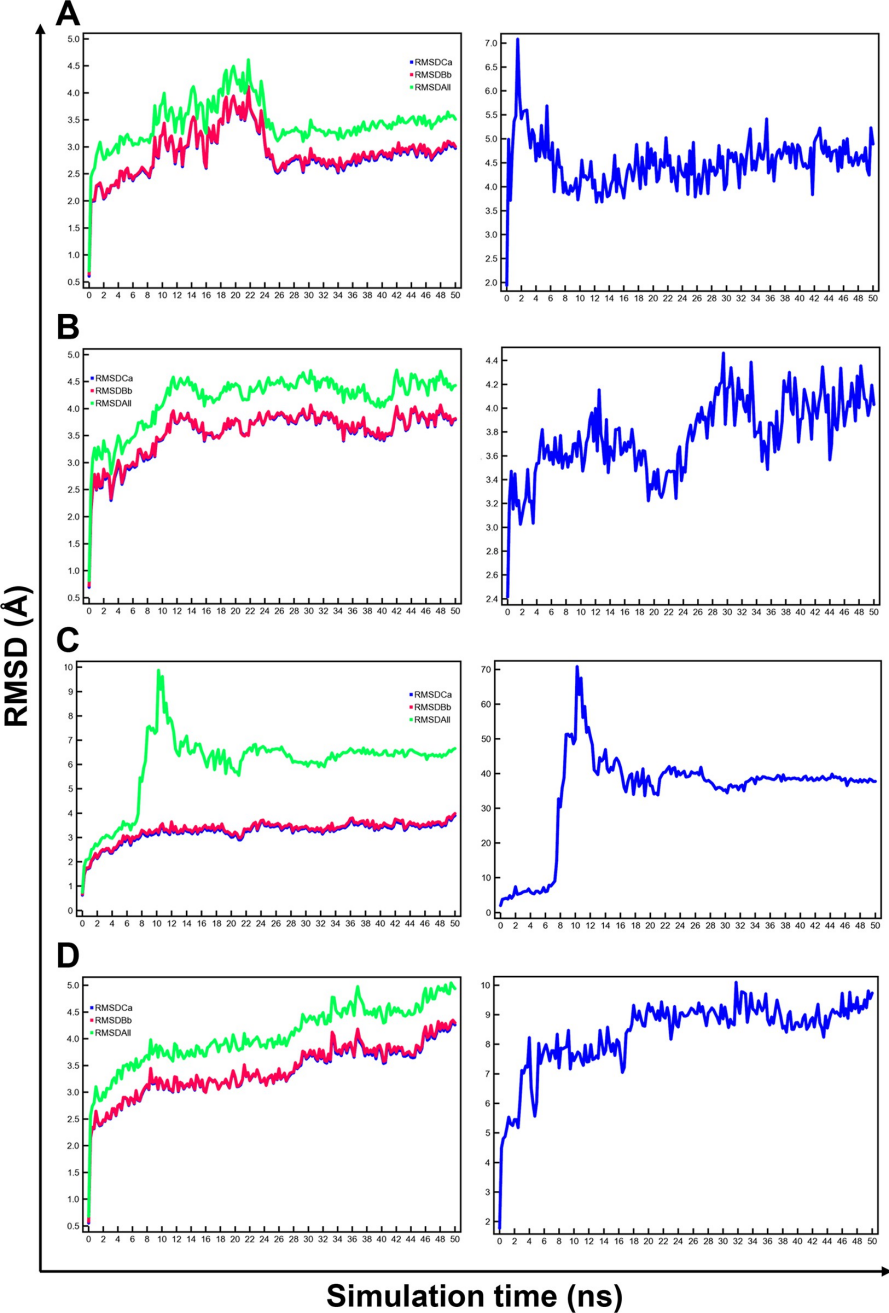
Stability assessment of two SBATs and two ASBTs docking with compound 9806452. (A) OF-CsSBAT, (B) IF-CsSBAT, (C) OF-HsASBT, and (D) IF-HsASBT. (Left panel) RMSD values of Cα (RMSDCa in blue), backbone (RMSDBb in red), and all heavy atoms (RMSDAll in green) from each initial structure during 50 ns simulation time. (Right panel) The RMSD pattern of the compound within each complex.

Prior to binding free energy estimations, the stability of MD simulations was investigated through the inspection of structural properties. All heavy atoms of both OF-CsSBAT and IF-CsSBAT showed relatively stable RMSD values at 3.5 Å and 4.5 Å on average (Fig 7A and 7B), respectively, indicating a delay on their stabilization into binding site, whereas OF-HsASBT displayed structural fluctuations during the first 12 ns and IF-HsASBT remained unstable (Fig 7C and 7D). Overall, these results suggest that 24 ns for OF-CsSBAT and 12 ns for the remainder are suitable equilibration time and, therefore, the last 26 ns and 38 ns MD simulation trajectories were used to calculate mean ΔE_bind_ values using the MM/PBSA method.

To better understand the binding free energies of compound 9806452-protein complex, we analyzed the MM/PBSA energy components (Table 6). MM/PBSA results show that OF-CsSBAT and IF-CsSBAT have better ΔE_bind_ values (42.3 kcal/mol and 88.7 kcal/mol) than OF-HsASBT and IF-HsASBT (38.5 kcal/mol and 22.5 kcal/mol). In this regard, movement of compound 9806452 revealed that OF-CsSBAT and IF-CsSBAT was stabilized around at RMSD values of 4.5 and 4.0 Å (Fig 7A and 7B), whereas OF-HsASBT and IF-HsASBT reached RMSD values of 70 Å and 10 Å, respectively (Fig 7C and 7D).

**Table 6:** Binding free energy and its components during MD simulation.

Systems^a^	CsSBAT		HsASBT	
	OF	IF	OF	IF
ΔE_bind_ (kcal/mol)^b^	42.3	88.7	38.5	22.5
E_potProt_ (kcal/mol)	–1657.1	–1678.6	–566.8	–535.7
E_solvProt_ (kcal/mol)	–2009.6	–2163.0	–2194.9	–2218.9
E_potLigand_ (kcal/mol)	38.7	21.6	18.9	–0.5
E_solvLigand_ (kcal/mol)	–45.6	–35.6	–33.4	–36.2
E_potComplex_ (kcal/mol)	–1684.8	–1727.0	–561.2	–583.8
E_solvComplex_ (kcal/mol)	–2031.1	–2217.4	–2253.5	–2230.1

^a^In all systems, 12–50 ns simulation was used for calculation of MM/PBSA except for OF-CsSBAT for 24–50 ns.

^b^More positive ΔE_bind_ values indicate better binding.

All abbreviations are defined in “Molecular dynamics (MD) simulation and binding free energies” section of Methods.

### Limitations and countermeasures of CsSBAT-targeted virtual screening

It should be noted that experimental validation is necessary to derive any meaning from this computational study. Protein-ligand docking could result in a high false positive prediction due to several limitations, such as unreliability of scoring methods, and lack of protein flexibility and water molecules during the simulation.

We attempted to address these limitations. First, we used the experimentally-characterized ligand-bound structures (4n7x and 3zuy) to find the geometries of more accurate binding pockets. Then, we validated our virtual docking protocol by re-docking of the substrate between experimental binding mode and predicted mode. Second, docking values were not used as a measure for compound activity, but rather we applied the relative affinities between OF- and IF-conformations, and between parasite and human. Then, we applied MM/PBSA method that accounts for the influence of solvents on the stability of the protein-ligand complex. Third, we did not incorporate water molecules in our docking simulations due to the computational complexity involved. Instead, MD simulation was applied because it enables protein-ligand complex to be simulated in water.

## Conclusions

*Clonorchis sinensis* sodium-bile acid cotransporter (CsSBAT) is indispensable for the worm’s survival in the bile duct. Inhibition of the bile transporters may perturb bile acid transport and is proven to be detrimental to *C*. *sinensis*. We identified the inhibitory compounds targeting the bile acid-binding pockets in CsSBAT, based on the physiological essence of crucial importance using structure-based drug discovery approach. First, two PubChem compounds were selected by applying the Lipinski’s rule of five. Furthermore, we devised a feasible rational screening strategy to search inhibitor candidates with molecular weight greater than 500 Da. Two large inhibitory compounds were selected with expectation of tight binding to CsSBAT. Of these candidates, compound 9806452 revealed the least oral toxicity and hepatotoxicity that may enhance its druggability. Collectively, compound 9806452 was proposed as a putative inhibitor of CsSBAT, deserving further *in vitro* and *in vivo* evaluation toward anthelminthic development.

## Supporting information

S1 Fig Homologous template searches for building tertiary structure of CsSBAT. Termini of CsSBAT were both hypothetical polypeptides. CsSBAT model was built on residues 185–492, based on two reliable templates (PDB ID: 3zuy_A and 4n7x_A).
(TIF)

S2 Fig Potential Na^+^- and taurocholate-binding sites of CsSBAT. All ligands and interaction were predicted using COACH [50] except for Na3 site (A). Na^+^-binding sites (B, C), Na1 site (B) and Na2 site (C). Putative Na3 site (D) as suggested by Alhadeff et al. [71]. The consensus residues forming the binding site are presented in stick mode and labeled. Side-chain oxygen, nitrogen, and sulfur atoms are indicated in red, blue, and yellow, respectively. Na^+^ ion is depicted as a purple ball and taurocholate substrate as stick mode.
(TIF)

S3 Fig Docked pose of taurocholate with OF-CsSBAT and IF-CsSBAT. Schematic interactions of taurocholate with OF-CsSBAT (A) and IF-CsSBAT (B) were plotted using LigPlot+ v1.4.3. Residues in close contact with a compound are visualized with 2D diagrams. Amino acid residues involved in hydrophobic interactions are presented as red spoked arcs. Residues contributing to hydrogen bonds are depicted in green and atomic distance (Å) is given in green number. The binding modes of compounds were obtained from the AutoDock Vina v1.1.2.
(TIF)

S1 Table Binding free energies and drug-like properties of 19 compounds from virtual screening against OF-SBAT and IF-SBAT of *Clonorchis sinensis* using MTiOpenScreen. (DOCX)

S2 Table Binding energy and toxicity risk of 25 compounds with high molecular weight (Mr > 500 Da) against OF-/IF-SBAT of *Clonorchis sinensis* and OF-/IF-ASBT of *Homo sapiens* using AutoDock Vina v1.1.2. (DOCX)
