# Supplementary figures and images for: Structure-based virtual screening and molecular dynamics of potential inhibitors targeting sodium-bile acid co-transporter of carcinogenic liver fluke Clonorchis sinensis
Source: PLoS Negl Trop Dis. 2022 Nov 9;16(11):e0010909. doi: 10.1371/journal.pntd.0010909 (PMC9645658; doi:10.1371/journal.pntd.0010909)

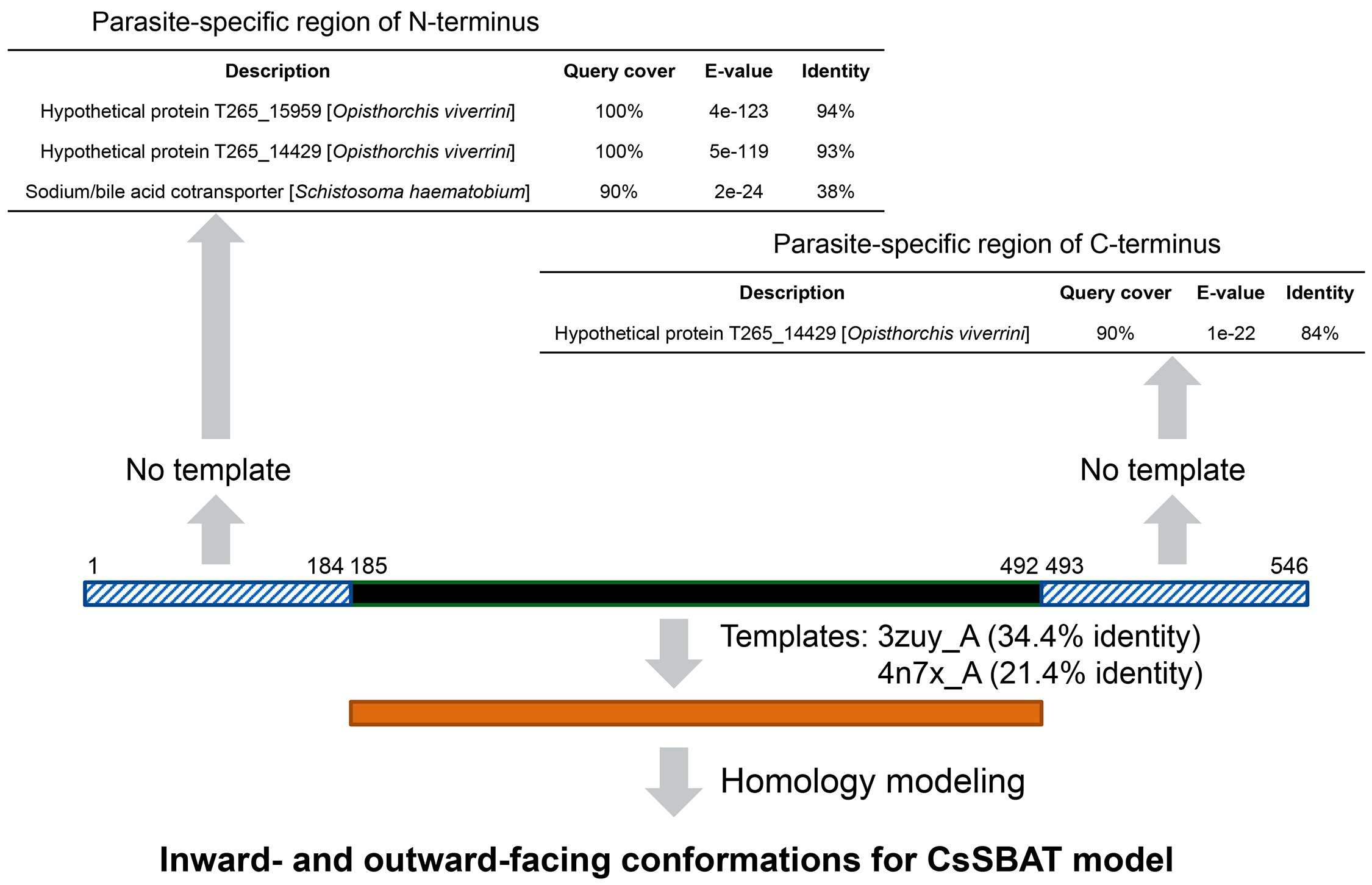

Supplement: S1 Fig — Termini of CsSBAT were both hypothetical polypeptides. CsSBAT model was built on residues 185–492, based on two reliable templates (PDB ID: 3zuy_A and 4n7x_A). (TIF) [file pntd.0010909.s001.tif]

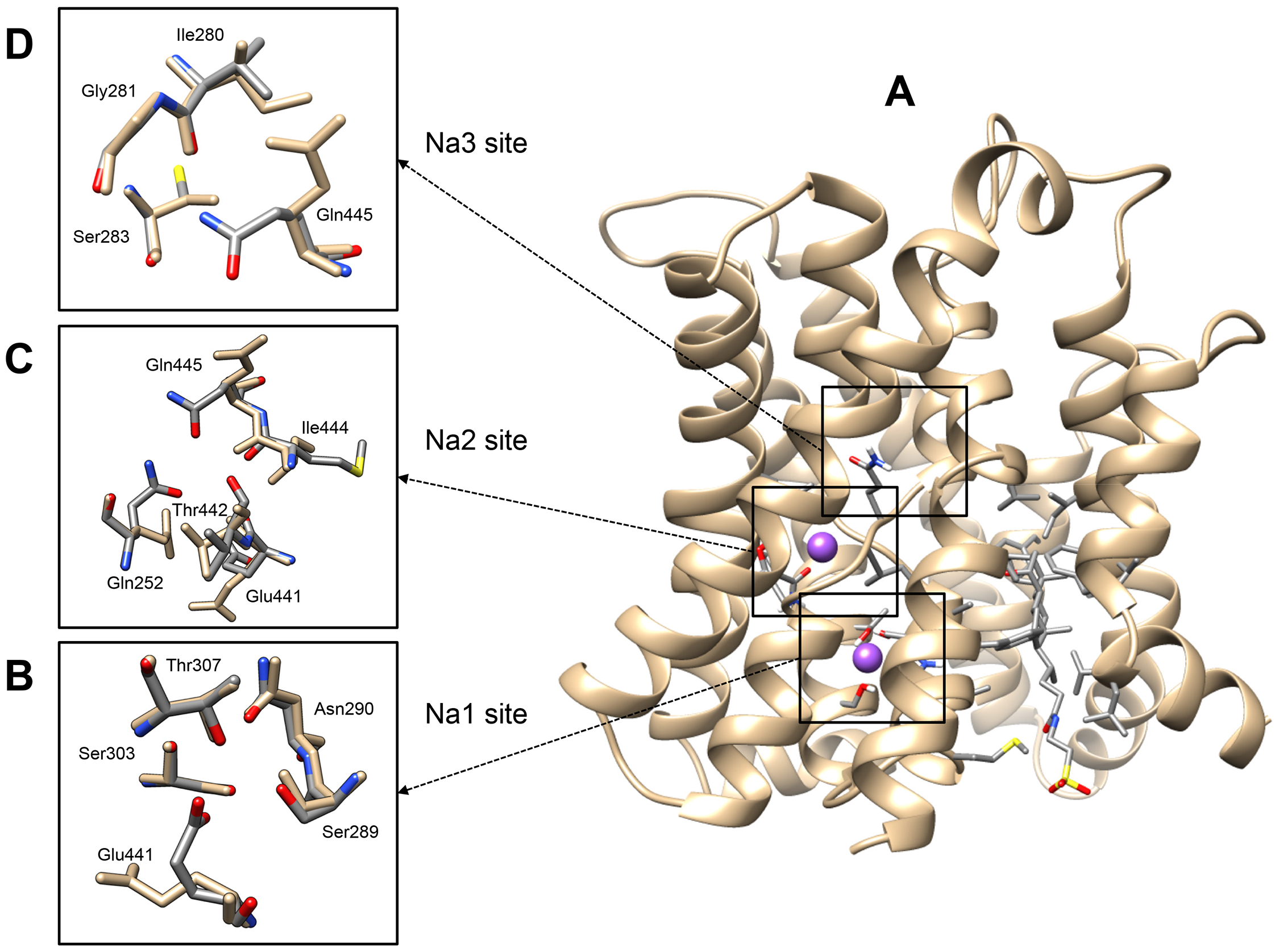

Supplement: S2 Fig — All ligands and interaction were predicted using COACH [50] except for Na3 site (A). Na+-binding sites (B, C), Na1 site (B) and Na2 site (C). Putative Na3 site (D) as suggested by Alhadeff et al. [71]. The consensus residues forming the binding site are presented in stick mode and labeled. Side-chain oxygen, nitrogen, and sulfur atoms are indicated in red, blue, and yellow, respectively. Na+ ion is depicted as a purple ball and taurocholate substrate as stick mode. (TIF) [file pntd.0010909.s002.tif]

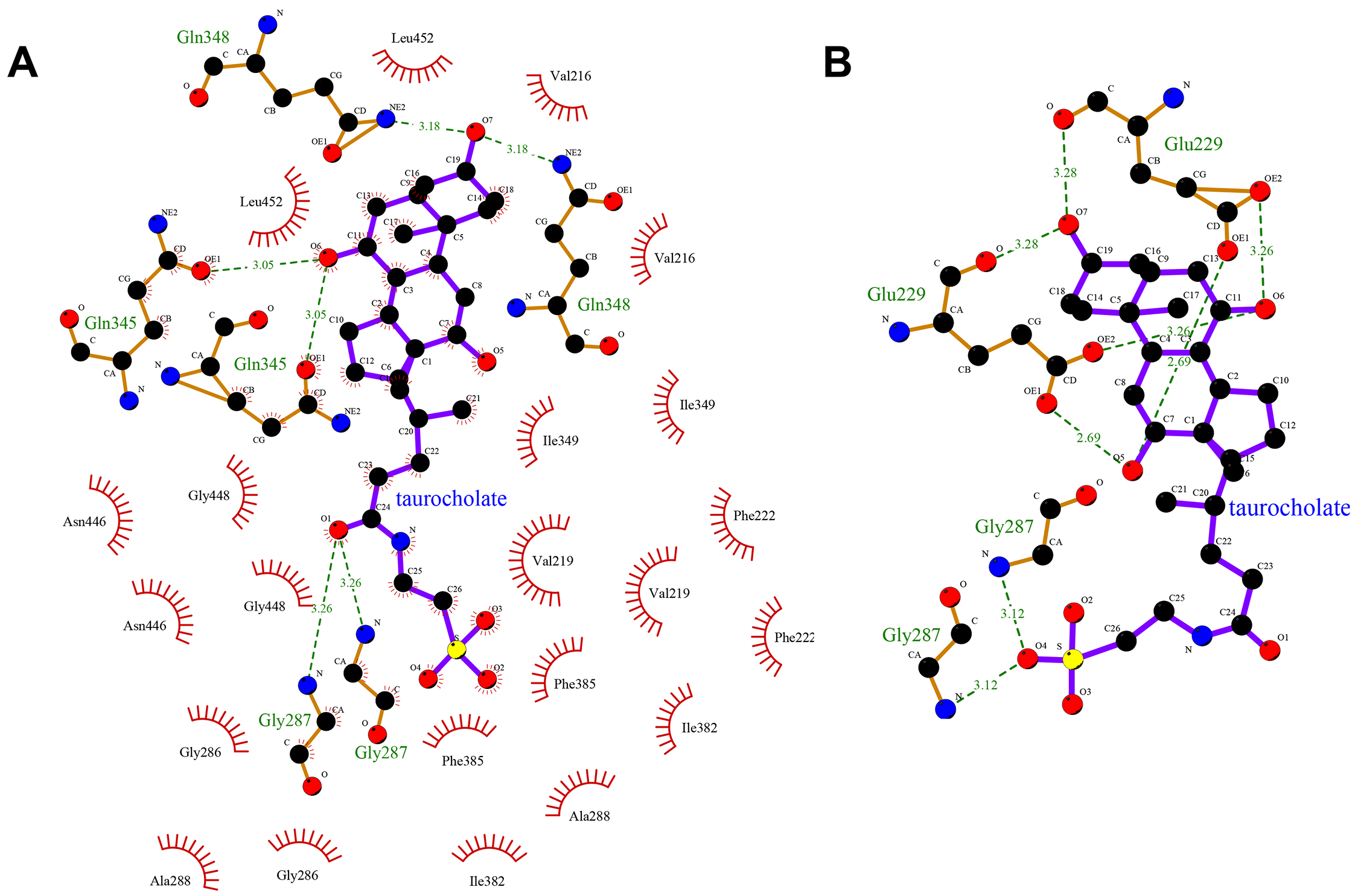

Supplement: S3 Fig — Schematic interactions of taurocholate with OF-CsSBAT (A) and IF-CsSBAT (B) were plotted using LigPlot+ v1.4.3. Residues in close contact with a compound are visualized with 2D diagrams. Amino acid residues involved in hydrophobic interactions are presented as red spoked arcs. Residues contributing to hydrogen bonds are depicted in green and atomic distance (Å) is given in green number. The binding modes of compounds were obtained from the AutoDock Vina v1.1.2. (TIF) [file pntd.0010909.s003.tif]
