# Supplementary material for: Structure-based virtual screening and molecular dynamics of potential inhibitors targeting sodium-bile acid co-transporter of carcinogenic liver fluke Clonorchis sinensis
Source: PLoS Negl Trop Dis. 2022 Nov 9;16(11):e0010909. doi: 10.1371/journal.pntd.0010909 (PMC9645658; doi:10.1371/journal.pntd.0010909)
Supplement: S1 Table — (DOCX) [file pntd.0010909.s004.docx]

**S1 Table. Binding free energies and drug-like properties of 19 compounds from virtual screening against OF-SBAT and IF-SBAT of *Clonorchis sinensis* using MTiOpenScreen.**

| **PubChem ID** | **Binding energy**  **(kcal/mol)** | |  | **Drug-like properties** | | | | | |  | **Hydrogen bond** | |
| --- | --- | --- | --- | --- | --- | --- | --- | --- | --- | --- | --- | --- |
|  | **OF** | **IF** |  | **nRot** | **HBA** | **HBD** | **LogP** | **Mr** | **TPSA** |  | **OF** | **IF** |
| 49734421 | −10.9 | −9.3 |  | 4 | 7 | 1 | 2.3 | 396.5 | 94.8 |  | 1 | 1 |
| 7972293 | −10.7 | −8.7 |  | 5 | 6 | 1 | 3.1 | 395.8 | 75.4 |  | 0 | 0 |
| 56318972 | −10.4 | −8.2 |  | 6 | 7 | 0 | 3.0 | 436.6 | 92.4 |  | 2 | 0 |
| 104223076 | −10.3 | −9.1 |  | 8 | 6 | 1 | 2.9 | 401.5 | 81.1 |  | 0 | 1 |
| 124948115 | −10.3 | −9.3 |  | 5 | 6 | 1 | 2.8 | 397.5 | 67.6 |  | 2 | 0 |
| 26536289 | −10.3 | −8.4 |  | 3 | 6 | 0 | 3.2 | 345.3 | 69.1 |  | 0 | 0 |
| 85268567 | −10.2 | −8.5 |  | 5 | 6 | 0 | 1.8 | 382.3 | 60.8 |  | 2 | 1 |
| 22407574 | −10.1 | −8.6 |  | 7 | 5 | 1 | 3.9 | 364.4 | 70.1 |  | 1 | 0 |
| 49718517 | −10.0 | −8.3 |  | 7 | 6 | 1 | 3.5 | 365.4 | 77.3 |  | 1 | 1 |
| 865205 | −9.9 | −8.4 |  | 1 | 4 | 0 | 3.6 | 266.3 | 71.3 |  | 1 | 0 |
| 49736001 | −9.8 | −8.4 |  | 5 | 6 | 1 | 2.8 | 427.5 | 114.7 |  | 0 | 1 |
| 24369200 | −9.7 | −8.7 |  | 6 | 5 | 1 | 3.9 | 387.9 | 64.6 |  | 1 | 1 |
| 124948721 | −9.7 | −8.1 |  | 5 | 7 | 0 | 2.3 | 383.5 | 64.7 |  | 2 | 0 |
| 26613646 | −9.7 | −8.7 |  | 3 | 5 | 1 | 1.8 | 359.4 | 66.1 |  | 1 | 0 |
| 26531450 | −9.7 | −8.2 |  | 2 | 2 | 0 | 3.6 | 248.3 | 30.2 |  | 2 | 0 |
| 49728725 | −9.6 | −8.2 |  | 5 | 5 | 1 | 3.6 | 378.5 | 74.9 |  | 0 | 0 |
| 26651178 | −9.6 | −8.2 |  | 4 | 5 | 2 | 2.4 | 369.5 | 63.4 |  | 1 | 1 |
| 49680193 | −9.5 | −8.4 |  | 8 | 5 | 2 | 3.8 | 416.5 | 67.4 |  | 1 | 1 |
| 24403006 | −9.4 | −8.8 |  | 6 | 6 | 1 | 3.9 | 429.4 | 81.1 |  | 1 | 1 |

Abbreviations: nRot, number of rotatable bonds; HBA, hydrogen bond acceptors; HBD, hydrogen bond donors; LogP, lipophilicity; Mr, molecular weight; TPSA, topological polar surface area.
