# Supplementary material for: Structure-based virtual screening and molecular dynamics of potential inhibitors targeting sodium-bile acid co-transporter of carcinogenic liver fluke Clonorchis sinensis
Source: PLoS Negl Trop Dis. 2022 Nov 9;16(11):e0010909. doi: 10.1371/journal.pntd.0010909 (PMC9645658; doi:10.1371/journal.pntd.0010909)
Supplement: S2 Table — (DOCX) [file pntd.0010909.s005.docx]

**S2 Table.** **Binding energy and toxicity risk of 25 compounds with high molecular weight (Mr > 500 Da) against OF-/IF-SBAT of *Clonorchis sinensis* and OF-/IF-ASBT of *Homo sapiens* using AutoDock Vina v1.1.2.**

|  |  |  | ***C. sinensis***  **(kcal/mol)** | | ***H. sapiens***  **(kcal/mol)** | | **Hydrogen bond** | | **Toxicity** | |
| --- | --- | --- | --- | --- | --- | --- | --- | --- | --- | --- |
| **PubChem ID** | **Mr** | **MF** | **OF** | **IF** | **OF** | **IF** | **OF** | **IF** | **Class** | **Target** |
| 441243 | 670.8 | C_38_H_50_N_6_O_5_ | −12.3 | −10.0 | −8.9 | −7.9 | 5 | 1 | 4 | no |
| 5281227 | 564.8 | C_40_H_52_O_2_ | −12.0 | −9.4 | −6.1 | −6.8 | 2 | n.a. | 6 | ANDR;AOFA;GCR;PRGR |
| 5481119 | 574.8 | C_35_H_47_FN_4_O_2_ | −11.8 | −10.1 | −8.8 | −9.0 | 2 | n.a. | 4 | DRD3 |
| 65463 | 505.9 | C_28_H_35_C_l3_N_2_ | −11.5 | −10.5 | −9.1 | −8.8 | 0 | n.a. | 5 | HRH1;OPRM |
| 3693566 | 612.6 | C_27_H_31_F_3_N_4_O_7_S | −11.5 | −10.3 | −9.1 | −8.9 | 6 | n.a. | 5 | OPRM |
| 5282483 | 507.4 | C_23_H_27_C_l2_F_3_N_2_OS | −11.3 | −9.6 | −8.3 | −8.2 | 4 | 6 | 3 | no |
| 4701 | 508.6 | C_31_H_32_N_4_O_3_ | −11.2 | -10.9 | −8.9 | −8.7 | 4 | 0 | 4 | no |
| 37393 | 500.4 | C_26_H_30_C_l2_F_3_NO | −11.1 | −9.3 | −7.9 | −7.7 | 2 | 0 | 5 | OPRM |
| 5311065 | 1069.2 | C_46_H_64_N_14_O_12_S_2_ | −10.8 | −9.7 | −8.1 | −8.5 | 4 | 8 | 3 | OPRM |
| 67356 | 510.4 | C_22_H_28_C_l2_F_3_N_3_OS | −10.7 | −9.4 | −8.0 | −8.0 | 4 | 6 | 3 | ANDR;DRD3;HRH1 |
| 11250029 | 569.7 | C_32_H_35_N_5_O_5_ | −10.5 | −9.9 | −8.9 | −8.9 | 2 | 4 | 4 | OPRM |
| 60923 | 587.2 | C_34_H_35_CIN_2_O_3_S | −10.4 | −10.7 | −9.0 | −9.1 | 0 | 0 | 4 | PGH1 |
| 4912 | 516.8 | C_31_H_48_O_2_S_2_ | −10.3 | −10.1 | −8.0 | −8.6 | 0 | 0 | 5 | AOFA;PGH1 |
| 16051933 | 1069.2 | C_46_H_64_N_14_O_12_S_2_ | −10.2 | −9.6 | −8.5 | −8.6 | 4 | 8 | 3 | OPRM |
| 37392 | 536.9 | C_26_H_31_C_l3_F_3_NO | −10.1 | −9.3 | −8.2 | −7.7 | 2 | 0 | 5 | OPRM |
| 644077 | 1084.2 | C_46_H_65_N_15_O_12_S_2_ | −10.1 | −9.3 | −7.7 | −8.3 | 4 | 6 | 3 | OPRM |
| 71420 | 513.5 | C_29_H_34_C_l2_N_2_O_2_ | −10.1 | −10.7 | −9.1 | −8.3 | 0 | 0 | 3 | AOFA;HRH1;OPRM |
| 27991 | 1069.2 | C_46_H_64_N_14_O_12_S_2_ | −10.0 | −9.7 | −7.8 | −8.6 | 2 | 10 | 3 | OPRM |
| 92727 | 628.8 | C_37_H_48_N_4_O_5_ | −10.0 | −10.0 | −9.0 | −8.7 | 4 | 0 | 5 | no |
| 9849616 | 522.3 | C_23_H_23_BrC_l2_N_4_O | −9.9 | −9.9 | −8.7 | −8.6 | 0 | n.a. | 4 | ADRB2;OPRM |
| 104849 | 500.2 | C_22_H_22_C_l4_N_4_O | −9.9 | −10.5 | −8.8 | −8.9 | 0 | n.a. | 4 | ADRB2;OPRM |
| 45375808 | 529.5 | C_22_H_29_FN_3_O_9_P | −9.7 | −9.7 | −8.4 | −8.4 | 2 | 4 | 6 | no |
| 656668 | 505.6 | C_28_H_31_N_3_O_6_ | −9.6 | −10.3 | −8.3 | −8.6 | 0 | n.a. | 3 | ANDR;AOFA;GCR;PGH1;PRGR |
| 3034011 | 523.4 | C_28_H_30_INO | −9.5 | −9.6 | −8.1 | −7.5 | 0 | n.a. | 4 | ANDR;AOFA;HRH1 |
| 9806452 | 512.7 | C_30_H_41_FN_2_O_4_ | −9.3 | −9.3 | −7.6 | −8.3 | 4 | 2 | 5 | no |

Abbreviations: Mr, molecular weight; MF, molecular formula; n.a., not available; ADRB2, adrenoceptor beta 2; ANDR, androgen receptor; AOFA, amine oxidase [flavin-containing] A; GCR, glucocorticoid receptor; PRGR, nuclear receptor subfamily 3 group C member 3; DRD3, dopamine receptor D3; PGH1, prostaglandin G/H synthase 1; HRH1, histamine receptor H1; OPRM, mu-type opioid receptor.
